# Pricing Mechanism Design for Centralized Pollutant Treatment with SME Alliances

**DOI:** 10.3390/ijerph13060622

**Published:** 2016-06-22

**Authors:** Yuyu Li, Bo Huang, Fengming Tao

**Affiliations:** 1 College of Computer and Information Science, Chongqing Normal University, Chongqing 400047, China; lyyjame@163.com; 2 College of Economics and Business Administration, Chongqing University, Chongqing 400044, China; 3 College of Mechanical Engineering, Chongqing University, Chongqing 400044, China; taofengming@cqu.edu.cn

**Keywords:** centralized pollutant treatment, pricing mechanism, small and medium-sized enterprises (SMEs), SME alliances, moral hazard, bargaining game

## Abstract

In this paper, we assume that a professional pollutant treatment enterprise treats all of the pollutants emitted by multiple small and medium-sized enterprises (SMEs). In order to determine the treatment price, SMEs can bargain with the pollutant treatment enterprise individually, or through forming alliances. We propose a bargaining game model of centralized pollutant treatment to study how the pollutant treatment price is determined through negotiation. Then, we consider that there is a moral hazard from SMEs in centralized pollutant treatment; in other words, they may break their agreement concerning their quantities of production and pollutant emissions with the pollutant treatment enterprise. We study how the pollutant treatment enterprise can prevent this by pricing mechanism design. It is found that the pollutant treatment enterprise can prevent SMEs’ moral hazard through tiered pricing. If the marginal treatment cost of the pollutant treatment enterprise is a constant, SMEs could bargain with the pollutant treatment enterprise individually, otherwise, they should form a grand alliance to bargain with it as a whole.

## 1. Introduction

Small and medium-sized enterprises (SMEs) are the main driving force for economic development all over the world, thanks to their flexibility and quick response to market demand changes. On the other hand, they are the main source of environmental pollution due to their backward production and pollutant treatment technologies, or a lack of capital for purchasing pollutant treatment equipment [1,2]. For example, in China, SMEs create over 60% of the GDP (Gross Domestic Product) and 80% of the employment and pay over 50% of the taxes but, at the same time, they are responsible for 60% of the economic losses caused by environmental pollution [3,4].

One of the main reasons why SMEs cause such significant damage to the environment is that most SMEs regard voluntary environmental activities as costly and unnecessary “extras” that endanger their competitiveness and detract resources from their core business, without offering any tangible benefits [5]. Meanwhile, most of the existent environment regulation policies, such as emission permits, emission taxes, and government subsidies, have little effect on SMEs, since they are established mainly for regulating the pollutant emissions of large-sized enterprises [6,7,8]. For example, Studer *et al.* found that the main barriers of SMEs in Hong Kong and many other countries with voluntary environmental initiatives are inadequate government policy and support, societal attitudes, and corporate culture [5].

Although with the improvement of environmental legislation and the increased public awareness of environmental protection issues, some SMEs are engaged in some environmental initiatives, the status quo is not satisfying. For example, Masurel found that improving working conditions is the most important reason why SMEs invest in environmental measures, as this improves their employees’ motivation and performance [9]. Jansson *et al.* found that SMEs committed to environmental sustainability see both market and entrepreneurial advantages in sustainability [10]. Brammer *et al.* found that besides legislation, strategic intent is the primary driving force of SMEs’ environmental management actions. However, medium sized firms perceive greater commercial payoffs from engagement with environmental management that arise from long-term financial benefits and increased market share, while small businesses perceive very few benefits of environmental management and their initiatives of committing to environmental activities are low. They believed that the lack of financial resources, managers’ values and knowledge are some of the most likely causes [11]. Additionally, there are two other causes: lack of advanced pollutant treatment technology and diseconomies of scale due to their small size. The former causes SMEs to fail to meet government pollutant treatment standards, and the latter causes them higher treatment costs. As a result, SMEs have very little initiative to participate in environmental activities [2].

Therefore, a pollutant treatment mode specially designed for SMEs has been widely adopted all over the world. Under this mode, several SMEs of a similar industry are centrally located in the same place, for example, an industrial park, and a professional pollutant treatment enterprise is responsible for treating all the pollutants generated by the SMEs’ production processes. Centralized pollutant treatment realizes the economies of scale in SME’s pollutant treatment, and solves the problem that SMEs are unable to afford the huge investment in pollutant treatment equipment due to their lack of financial resources. Thus, this centralized pollutant treatment mode achieves good performance in SMEs’ pollutant treatment, and has gained global impetus [12].

Most research on centralized pollutant treatment has concentrated on the necessity, feasibility, experience, and policy suggestions for carrying out centralized pollutant treatment. Roomratanapun applied the theory of diffusion and adoption to examine the acceptance of the centralized pollutant treatment project in Bangkok, used the contingent valuation method to analyze the willingness to pay for the project, and utilized the theory of environmental psychology to investigate the factors influencing acceptance of the centralized pollutant treatment project [13]. Yuan *et al.* examined the effectiveness of two operational strategies: a decentralized model and an innovative integrated model, which have been used for treating wastewater from the city’s dyeing industry in the Shengze industrial town of Suzhou City [14]. Chittock and Hughey identified the nine key features of a successful centralized pollutant treatment by reviewing some programs in Australia, Canada, Japan, the United Kingdom, and the United States [15]. Avram and Künhe used a medium-sized company selected from among 250,000 SMEs for an exploratory case study analysis to show the performance of centralized pollutant treatment [16]. He and He found that carrying out centralized pollutant treatment realizes the economies of scale in pollutant treatment for SMEs considering the characteristics of SMEs’ production and pollutant emission in China [17].

Furthermore, some scholars have studied how to design proper mechanisms to prevent SMEs’ stealth emissions, that is, firms that stealthily emit untreated pollutant into environment as this is illegal. Hu and Huang developed a regulation game model among government, SMEs and the pollution control company to study the optimal policies of pollutant emission regulation and pollution control under centralized pollution control [18]. Li proposed a game model among government, pollutant treatment enterprises, and SMEs to design the government’s optimal regulatory mechanism, pollutant treatment enterprise’s treatment policies, and the SMEs’ production and emission policies [19].

The research above does not solve the key problem in any centralized pollutant treatment scheme, *i.e.*, pollutant treatment pricing. Under the current centralized pollutant treatment, SMEs have no right to select the centralized pollutant treatment enterprise, which is assigned by the government. Therefore, the pollutant treatment enterprise will certainly use its natural perfect monopoly to set a high treatment price, which may cause SMEs to quit centralized pollutant treatment. In order to settle the problem, some governments have to regulate the pricing behavior of the pollutant treatment enterprise at the cost of giving it a preference, such as the practice in the Zhejiang Songyang Industrial Zone. Cui *et al.* reached the same conclusion through a Stackelberg game model between SMEs and the pollutant treatment enterprise [20].

Our suggestion is that government should not assign any pollutant treatment enterprise to SMEs; instead, SMEs can select any pollutant treatment enterprise recommended by the government and fairly negotiate with them on the price. As a result, the centralized pollutant treatment will be carried out more effectively to improve the utilization efficiency and maximize the social welfare.

The most popular model used to analyze the negotiation in cooperation is the Nash Bargain Model proposed by Nash in 1953 [21]. Since then, it has been further developed and widely used in many economic issues based on negotiations. For example, McGuire and Staelin investigated the effect of product substitutability on Nash equilibrium distribution structures in a duopoly supply chain [22]. Nagarajan and Bassok studied the bargaining framework in a decentralized supply chain consisting of a single assembler and multiple suppliers, and how they share the total profit through negotiation [23]. Escapa and Gutierrez quantified how the potential gains derived from environmental cooperation among countries would be distributed among them [24]. Solow and Krautmann studied how an elite sports player and a team determine the player’s location and salary [25]. Therefore, in this paper, we use the Nash Bargain Model to analyze the pricing policies for centralized pollutant treatment.

This paper has two main contributions: firstly, we propose a bargaining game model to study how the pollutant treatment prices are determined through negotiation between SMEs and a professional pollutant treatment enterprise treating the pollutants of all SMEs and we study SMEs’ ally policies, *i.e.*, under what conditions should they form what kind of alliance to negotiate with the pollutant treatment enterprise. Secondly, we study how the pollutant treatment enterprise prevents SMEs’ moral hazard of emitting pollutants in breach of their agreement on the emission quantity through designing a pricing mechanism. We analyze the effect of the pollutant treatment enterprise’s bargaining power, decision rights on negotiation sequence, marginal cost of pollutant treatment, and the fluctuation of product’s selling price on SMEs’ negotiation and alliance policies.

## 2. The Negotiation Model

Let us assume there are *n* SMEs in the same region, who manufacture the same kind of products with few differences on quality and performance. Therefore, SMEs compete with each other by lower price, and the product prices *P* of all SMEs are the same [26]. The production cost *C_i_* of a SME product *i* (*i* = 1, 2, …, *n*), is the function of its production quantity *q_i_*, *i.e.*, $C_{i}=\;C_{i}\left(q_{i}\right)$, which satisfies $C_{i}\left(0\right)=0,$ $C_{i}^{\prime }\left(q_{i}\right)>0$, $C_{i}^{\prime \prime }\left(q_{i}\right)>0$. Pollutants are generated in the production process, and the pollutant quantity of SME *i* is the function of its production quantity, *i.e.*, $E_{i}=\;E_{i}\left(q_{i}\right)$, which satisfies $E_{i}\left(0\right)=0$ and $E_{i}^{\prime }\left(q_{i}\right)>0$.

The government centralizes SMEs into an industrial park, which is an established model in many countries because it generates considerable economic and environmental benefits due to an institutional system that effectively combines top-down and bottom-up approaches, as well as local stakeholders from businesses, governments, and research organizations [27]. Then, a professional pollutant treatment enterprise is selected to treat all the pollutant generated by all SMEs. As the SMEs are centralized together, and online monitoring technology is very mature and widely adopted by governments, the government can easily monitor the emission behavior of SMEs and the pollutant treatment enterprises. Additionally, the punishments for stealthy emissions are very heavy. Therefore, we assume that SMEs and pollutant treatment enterprises will not emit stealthily.

The pollutant treatment enterprise’s cost is the function of the pollutant quantity it received, *i.e.*, $C_{e}=C_{e}\left(\sum \text{​}E_{i}\right)$, which satisfies $C_{e}\left(0\right)=0$, $C_{e}^{\prime }\left(\sum \text{​}E_{i}\right)>0$ and $C_{e}^{\prime \prime }\left(\sum \text{​}E_{i}\right)\leq 0$. In other words, the cost of cleaning an additional pollutant emission decreases as the quantity of emissions increases, which is the popular case both in theory and practice [28,29]. The pollutant treatment enterprise charges SME *i* (*i* = 1, 2, …, *n*), *P_i_E_i_* for pollutant treatment, where *P_i_* is the treatment price per unit pollutant.

The treatment price per unit pollutant *P_i_* is determined by the SMEs and the pollutant treatment enterprises through negotiation. In order to enhance their bargaining power, SMEs can choose to form alliances *s_j_* to negotiate, where, $s_{j}\subseteq N$ and *N* = {1, 2, …, *n*} is the set of all SMEs. There are two special kinds of alliance, one of which is a grand alliance of all SMEs, the other one of which is *n* alliances formed by *n* SMEs individually. The reader, without any loss of generality, may substitute a SME by an alliance of only itself. Therefore, *θ*, the number of SME alliances satisfies 1 ≤ *θ* ≤ *n*. We number the SME alliances according to the negotiation sequence, *i.e.*, the alliance *s_j_* is the *j^th^* alliance with whom the pollutant treatment enterprise negotiates. Furthermore, we assume that the pollutant treatment enterprise has the ability of determining the negotiation sequence.

As the negotiation process is sequential, at any point in time the pollutant treatment enterprise negotiates with only one SME alliance. The negotiation between the pollutant treatment enterprise and any SME alliance is captured by what we call the basic negotiation process:

(1) $$\text{max}{\left(\pi_{e}-d_{e}\right)}^{\alpha_{e,m}}\;{\left(\pi_{m}-d_{m}\right)}^{\beta_{e,m}},\;m\;=\;s_{j},\;j\;=\;1,2,\ldots ,\theta \;s.t.\;\left(\pi_{e},\;\pi_{m}\right)\;\geq \;\left(d_{e},d_{m}\right),\;\pi_{e}\;+\;\pi_{m}\;\leq \;\Pi_{j}$$

where $\Pi_{j},\;j=1,2,\ldots ,\theta$ is the total profit of all member of the alliance coalition *s_j_* and the treatment enterprise, and is allocated at the *j^th^* negotiation. Obviously, $\Pi_{j}$ satisfies:

(2) $$\Pi_{j}\;=\;p\underset{i\in s_{j}}{\sum }q_{i}\;-\;\left[C_{e}\;\left(Q_{j}+\underset{i\in s_{j}}{\sum }E_{i}\right)\;-\;C_{e}\left(Q_{j}\right)\right]-\underset{i\in s_{j}}{\sum }C_{i}\;\left(q_{i}\right),\;j\;=\;1,2,\ldots ,\theta$$

where $p\sum_{i\in s_{j}}q_{i}$ and $\sum_{i\in s_{j}}C_{i}\left(q_{i}\right)$ are the total sales revenue and total production cost of *s_j_* respectively, $C_{e}\;\left(Q_{j}+\sum_{i\in s_{j}}E_{i}\right)\;-\;C_{e}\left(Q_{j}\right)$ is the difference between the cost of treating pollutant from *s*_1_ through *s_j_* and that of treating pollutant from *s*_1_ through *s_j–_*_1_, *i.e.*, the cost of treating pollutant from *s_j_*, and *Q_j_*, *j* = 1, 2, …, *θ*, is the total pollutant emitted from SMEs’ alliances before *s_j_*, *i.e.*, *s*_1_ through *s_j–_*_1_. Obviously, $Q_{j}=\;\sum_{I=1}^{j-1}\sum_{i\in s_{I}}E_{i}$.

The terms *d_e_* and *d_m_* are the profits individually obtained by the pollutant treatment enterprise and SME alliance *s_j_* when they fail to reach an agreement. Obviously, only when $\left(\pi_{e},\;\pi_{m}\right)\;\geq \;\left(d_{e},d_{m}\right)$, *i.e.*, the pollutant treatment enterprise and SME alliance *s_j_* obtain more profits from centralized pollutant treatment, are they willing to take part in it. Simplifying the model, we further assume *d_e_* = *d_m_* = 0 according to Muthoo [30]. *α_e,m_* and *β_e,m_* are the representative of the pollutant treatment enterprise and SME alliance *s_j_*, respectively, which satisfy *α_e,m_* + *β_e,m_* = 1. Then, we can get that at the *j^th^* negotiation, the pollutant treatment enterprise obtains *α_e,m_*∏*_j_*, and SMEs’ alliance *s_j_* obtains *β_e,m_*∏*_j_*, which is shared proportionally by the SMEs in *s_j_*.

Before negotiation, the pollutant treatment enterprise and SME alliances commit to accepting no less than $\bar{\pi }$*_l_*, *l* = *e*, *s*_1_, …, *s_θ_*, or else they will quit the centralized pollutant treatment. However, these commitments are partial, and they can revoke their commitments at a cost of *c_l_*, which may be a loss of credibility or reputation. Muthoo assumes a linear revoking cost, given by:

(3) $$c_{l}=\{\begin{array}{ll} 0 & {\bar{\pi }}_{l}\leq \pi_{l}\\ k_{l}\left({\bar{\pi }}_{l}-\pi_{l}\right) & {\bar{\pi }}_{l}>\pi_{l} \end{array},\;l=e,s_{1},\ldots ,s_{\theta },$$

where, *k_l_* is the revocation cost parameter, which satisfies *k_l_* > 0.

Equation (3) means that the revoking cost is zero if the allocation *π_l_* obtained by the pollutant treatment enterprise or a SME alliance is more than $\bar{\pi }$*_l_*, while the revocation cost is *c_l_* = *k_l_*($\bar{\pi }$*_l_* – *π_l_*) if it revokes its commitments by accepting an allocation *π_l_* less than $\bar{\pi }$*_l_*. The less of the allocation it accepts, the bigger its revocation cost is.

The unique Nash equilibrium of the commitments at the *j^th^* negotiation is:

(4) $$\left({\bar{\pi }}_{e}^{*},{\bar{\pi }}_{m}^{*}\right)=\left(\pi_{e}^{*},\pi_{m}^{*}\right)=\left(\frac{\left(1+k_{e}\right)\Pi_{j}}{2+k_{e}+k_{m}},\frac{\left(1+k_{m}\right)\Pi_{j}}{2+k_{e}+k_{m}}\right),\;m=s_{j},\;j=1,2,\ldots ,\theta .$$

From Equations (3) and (4), we can find that the allocation obtained by the pollutant treatment enterprise or a SME alliance, which equates its partial commitments, is increasing in its revocation cost while decreasing in the corresponding cost of its opponent. The relationships between *k_l_* and *α_e,m_*, *β_e,m_* (*m = s_j_*, *j* = 1, 2, …, *θ*) are as follows:

(5) $$\alpha_{e,m}=\left(1+k_{e}\right)/\left(2+k_{e}+k_{m}\right),\;m=s_{j},\;j=1,2,\ldots ,\theta$$

(6) $$\beta_{e,m}=\left(1+k_{m}\right)/\left(2+k_{e}+k_{m}\right),\;m=s_{j},\;j=1,2,\ldots ,\theta$$

## 3. Model Analyses

### 3.1. The Pricing Policy When SMEs Bargain Individually

When SME *i* (*i* = 1, 2, …, *n*) bargains with the pollutant treatment enterprise individually, the SME alliance *s_j_* (*j* = 1, 2, …, *n*) consists of only SME *i*, itself (*i* = *j*). The profits of the pollutant treatment enterprise and SME *i*, which is the SME alliance *s_j_*, are as follows:

(7) $$\pi_{e}\left(P_{i},q_{i}\right)=P_{i}E_{i}-\left[C_{e}\left(Q_{j}+E_{i}\right)-C_{e}\left(Q_{j}\right)\right],\;i=j=1,2,\ldots ,n$$

(8) $$\pi_{i}\left(P_{i},q_{i}\right)=Pq_{i}-C_{i}-P_{i}E_{i},\;i=j=1,2,\ldots ,n$$

At the *j^th^* (*j* = 1, 2, …, *n*) negotiation, the pollutant treatment enterprise and SME *i* (*i* = *j*) firstly make a decision on *q_i_*, the product quantity of SME *i*, aiming at maximizing their total profit. Solving ∂∏*_j_*/∂*q_i_* = 0, we can get the optimal product quantity $q_{i}^{*}$, which is the solution of the following equation:

(9) $$P-C_{i}^{\prime }\left(q_{i}\right)-C_{e}^{\prime }\left(Q_{j}+E_{i}\right)E_{i}^{\prime }\left(q_{i}\right)=0.$$

Substituting $q_{i}^{*}$ (*i* = *j* = 1, 2, …, *n*) into Equations (2), (7), and (10), we can get the optimal total profit allocated at the *j^th^* negotiation $\prod_{j}^{*}$, the pollutant treatment enterprise profit $\alpha_{e,m}\prod_{j}^{*}$, and the profit of SME *i* $\beta_{e,m}\prod_{j}^{*}$, where *m* = *s_j_*. Now, we can obtain Proposition 1 as follows:

**Proposition 1.**  The profits of the pollutant treatment enterprise and SME *i* (*i* = *j* = 1, 2,…, n) obtained at the *j^th^* negotiation are, respectively, $\alpha_{e,m}\prod_{j}^{*}$ *and* $\beta_{e,m}\prod_{j}^{*}$ *where m = s_j_.*

**Proof of Proposition 1.**  The proof of Proposition 1 can be obtained from the above analysis.

**Proposition 2.**  SMEs take part in bargaining to share bigger profits by moving backward in the negotiation sequence.

**Proof of Proposition 2.**  See Appendix.

**Proposition 3.**  SME *i (i = j =* 1, 2, …, *n)* makes a payment $∆\pi_{i}^{*}=\beta_{e,m}(\Pi_{j}^{*}-\Pi_{1}^{*}$) to the pollutant treatment enterprise for the right of bargaining at the *j^th^* negotiation, where *m = s_j_.*

**Proof of Proposition 3.**  See Appendix.

From Propositions 2 and 3, we can find that SMEs obtain extra profits by moving backward in the negotiation sequence, while the right of determining the negotiation sequence is owned by the pollutant treatment enterprise. Therefore, the pollutant treatment enterprise will force SMEs to pay the whole extra profits for the position in the negotiation sequence, which causes SMEs to only obtain the profits in the first negotiation. Now, we can obtain Proposition 4 as follows:

**Proposition 4.**  If SMEs bargain individually, the final profits of all SMEs *i (i = j =* 1, 2, …, *n)* are $\beta_{e,m}\Pi_{1}^{*}$, while the final profit of the pollutant treatment enterprise is $\sum (\Pi_{j}^{*}-\beta_{e,m}\Pi_{1}^{*})$, where *m* = *s_j_*.

**Proof of Proposition 4.**  See Appendix.

Substituting $\beta_{e,m}\Pi_{1}^{*}$ into Equation (8), we can get the optimal treatment price per unit pollutant $P_{i}^{*}$ which the pollutant treatment enterprise charges SME *i* (*i* = 1, 2, …, *n*) as follows:

(10) $$P_{i}^{*}=\frac{Pq_{i}^{*}-C_{i}\left(q_{i}^{*}\right)-\beta_{e,m}\Pi_{1}^{*}}{E_{i}\left(q_{i}^{*}\right)},\;i=j=1,2,\ldots ,n,\;m=s_{j}$$

Obviously, the pollutant treatment enterprise can make contracts with SMEs on the treatment price per unit pollutant, but it cannot make contracts on the SMEs’ production quantities and pollutant emissions. Therefore, SMEs will not produce the quantities maximizing the pollutant treatment enterprise’s total profit, but maximizing their own profits. Solving ∂*π_i_*/∂*q_i_* = 0, (*i* = 1, 2, …, *n*) we can get the real production quantities of SME *i*, ${\bar{q}}_{i}$, are as follows:

(11) $$P-C_{i}^{\prime }\left(q_{i}\right)-P_{i}^{*}E_{i}^{\prime }\left(q_{i}\right)=0,\;i=1,2,\ldots ,n$$

Comparing Equations (9) and (11), we can find that ${\bar{q}}_{i}\;=\;q_{i}^{*}$ only if $P_{i}^{*}=C_{e}^{\prime }\left(Q_{j}\;+\;E_{i}\right)$, which is obviously a very specific circumstance. Generally, $P_{i}^{*}\neq C_{e}^{\prime }\left(Q_{j}+E_{i}\right)$, which means the real production quantities ${\bar{q}}_{i}$ are not the optimal ones $q_{i}^{*}$, and the real emissions $E_{i}\left({\bar{q}}_{i}\right)$ are not the optimal ones $E_{i}\left(q_{i}^{*}\right)$. As a result, the pollutant treatment enterprise will suffer a loss. Therefore, the pollutant treatment enterprise should design a mechanism to stimulate SMEs to produce and emit the quantities maximizing the total profit, and its profit, too. We suggest that the pollutant treatment enterprise should charge SMEs tiered prices to make them earn the maximum profits only if they produce and emit the quantities maximizing the total profiting. As a result, SMEs are stimulated. Now, we can obtain Proposition 5 as follows:

**Proposition 5.**  If SMEs bargain individually, the pollutant treatment enterprise charges them tiered prices as:

(12) $$P_{i}=\{\begin{array}{ll} P_{i}^{*}E_{i}\left({\bar{q}}_{i}\right)+F_{i}^{*} & E_{i}\left({\bar{q}}_{i}\right)<E_{i}\left(q_{i}^{*}\right)\\ P_{i}^{*}E_{i}\left({\bar{q}}_{i}\right) & E_{i}\left({\bar{q}}_{i}\right)=E_{i}\left(q_{i}^{*}\right)\\ P_{i}^{*}E_{i}\left(q_{i}^{*}\right)+\bar{P}E_{i}\left({\bar{q}}_{i}-q_{i}^{*}\right) & E_{i}\left({\bar{q}}_{i}\right)>E_{i}\left(q_{i}^{*}\right) \end{array},\;i=j=1,2,\ldots ,n$$

where $F_{i}^{*}=P_{i}^{*}E_{i}\left(q_{i}^{*}\right)-C_{e}\left(Q_{j}+E_{i}\left(q_{i}^{*}\right)\right)-\left[P_{i}^{*}E_{i}\left({\bar{q}}_{i}\right)-C_{e}\left(Q_{j}+E_{i}\left({\bar{q}}_{i}\right)\right)\right],\;{\bar{P}}_{i}>\frac{P\left({\bar{q}}_{i}-q_{i}^{*}\right)-\left[C_{i}\left({\bar{q}}_{i}\right)-C_{i}\left(q_{i}^{*}\right)\right]}{E_{i}\left({\bar{q}}_{i}\right)-E_{i}\left(q_{i}^{*}\right)}$.

**Proof of Proposition 5.**  See Appendix.

### 3.2. The Pricing Policy When SMEs Bargain in Alliance

From the above discussions, we can find that SMEs have to pay for their position in the negotiation sequence due to the lack of the ability to determine the sequence. As a result, they only get the profit that they can obtain in the first negotiation. Therefore, SMEs may bargain with the pollutant treatment enterprise as an alliance in order to increase their profits.

There is little difference in revocation cost and bargaining power among SMEs, as their scales, technologies and capabilities are very similar. Therefore, without loss of generality, we further assume that SMEs have the same bargaining power, and the bargaining power of an alliance is the average of that of its members, that is, *β_e,m_* = *β_e,i_* = *β*, *α_e,m_* = *α_e,i_* = *α*, where *i* = 1, 2, …, *n*, *j* = 1, 2, …, *θ*, *m* = *s_j_*. As a result, the SMEs in a same alliance allocate their alliance’s profits equally.

Let the number of members of alliance *s_j_* (*j* = 1, 2, …, *θ* ) be *η_j_*, we can get the profits of the pollutant treatment enterprise and the SMEs *i* of alliance *s_j_* as follows:

(13) $$\pi_{e}\left(P_{i},q_{i}\right)=P_{i}\eta_{j}E_{i}-\left[C_{e}\left(Q_{j}+\eta_{j}E_{i}\right)-C_{e}\left(Q_{j}\right)\right],\;i\in s_{j},\;j=1,2,\ldots ,\theta$$

(14) $$\pi_{i}\left(P_{i},q_{i}\right)=Pq_{i}-C_{i}-P_{i}E_{i},\;i\in s_{j},\;j=1,2,\ldots ,\theta$$

At the *j^th^* negotiation, the pollutant treatment enterprise and the SMEs’ alliance *s_j_* (*j* = 1, 2, …, *θ*) firstly determine *q_i_* (*i* ∈ *s_j_*), the product quantity of SME *i*, aiming at maximizing their total profit ∏*_j_* = *π_e_* + *η_j_π_i_*. Solving ∂∏*j*/∂*q_i_* = 0, we can get the optimal product quantity $q_{i}^{**}$ as the solution of the following equation.

(15) $$P-C_{i}^{\prime }\left(q_{i}\right)-C_{e}^{\prime }\left(Q_{j}+\eta_{j}E_{i}\right)E_{i}^{\prime }\left(q_{i}\right)=0$$

As SMEs’ capabilities are the same, their optimal product quantities $q_{i}^{**}$ (*i* ∈ *s_j_*, *j* = 1, 2, …, *θ*) are the same. Substituting $q_{i}^{**}$ into Equations (2), (13), and (14), we can get the optimal total profit allocated at the *j^th^* negotiation, $\Pi_{j}^{**}$, and the profits of the pollutant treatment enterprise and SMEs *i* of alliance *s_j_*, $\alpha \Pi_{j}^{**}$ and $\beta \Pi_{j}^{**}/\eta_{j}$.

**Proposition 6.**  If SMEs form more than one alliance, the pollutant treatment enterprise and SMEs *i* of alliance *s_j_ (i* ∈ *s_j_, j* = 1, 2, …, θ), obtain profits $\alpha \Pi_{j}^{**}$ and $\beta \Pi_{j}^{**}/\eta_{j}$ at the *j^th^* negotiation, respectively, and SMEs *i* pay the pollutant treatment enterprise $\beta \left(\Pi_{j}^{**}-\Pi_{1}^{**}\right)/\eta_{j}$ for the position in the negotiation sequence. As a result, their final profits are $\Pi_{j}^{**}-\beta \Pi_{1}^{**}$ and $\beta \Pi_{1}^{**}/\eta_{j}$ respectively.

**Proof of Proposition 6.**  See Appendix.

Proposition 6 shows that as long as SMEs form more than one alliance, SMEs still have to pay the pollutant treatment enterprise for the position in the bargaining sequence, which causes them to lose profits. Therefore, we obtain Proposition 7 as follows:

**Proposition 7.**  All SMEs should form a grand alliance to take part in the negotiation, in which the pollutant treatment enterprise and SMEs *i* (*i =* 1, 2, …, *n*), respectively obtain final profits *α*∏** and *β*∏**/n, where ∏** = pnq** – C_e_(nE**) – nC_i_(q**).

**Proof of Proposition 7.**  See Appendix.

Substituting *β*∏**/*n* into Equation (8), we can get the optimal treatment fee per unit pollutant, $P_{i}^{**}=P^{**}$, as follows:

(16) $$P^{**}=\frac{nPq^{**}-nC_{i}\left(q^{**}\right)-\beta \Pi^{**}}{nE_{i}\left(q^{**}\right)}$$

Obviously, the pollutant treatment enterprise still cannot make contracts on SMEs’ production quantities and pollutant emissions. Therefore, SMEs will produce the quantities maximizing their own profits, that is, their real product quantities $\tilde{q}$ are still the solution of Equation (11). Then, we can get that $\tilde{q}$ = q** only if $P^{**\;}=\;C_{e}^{\prime }\left(nE^{**}\right)$, so the pollutant treatment enterprise should charge SMEs tiered prices to make them earn the maximum profits only if they produce q**. Now, we can get Proposition 8 as follows:

**Proposition 8.**  If SMEs bargain in a grand alliance, the pollutant treatment enterprise charges them tiered prices as:

(17) $$P_{i}=\{\begin{array}{ll} P^{**}E_{i}\left(\tilde{q}\right)+F^{**} & E_{i}\left(\tilde{q}\right)<E_{i}\left(q^{**}\right)\\ P^{**}E_{i}\left(\tilde{q}\right) & E_{i}\left(\tilde{q}\right)=E_{i}\left(q^{**}\right)\\ P^{**}E_{i}\left(q^{**}\right)+\tilde{P}E_{i}\left(\tilde{q}-q^{**}\right) & E_{i}\left(\tilde{q}\right)>E_{i}\left(q^{**}\right) \end{array},\;i=1,2,\ldots ,n$$

where: $F^{**}=P^{**}E_{i}\left(q^{**}\right)-P^{**}E_{i}(\tilde{q)}-\frac{1}{n}\left[C_{e}\left(nE_{i}\left(q^{**}\right)\right)-C_{e}\left(nE_{i}\left(\tilde{q}\right)\right)\right]$, $\tilde{P}>\frac{P\left(\tilde{q}-q^{**}\right)-\left[C_{i}\left(\tilde{q}\right)-C_{i}\left(q^{**}\right)\right]}{E_{i}\left(\tilde{q}\right)-E_{i}\left(q^{**}\right)}$.

**Proof of Proposition 8.**  See Appendix.

### 3.3. The Alliance Policy of SMEs

From the above analysis, we can find that if $C_{e}^{\prime \prime }(\sum E_{i})<0$, the marginal pollutant treatment cost of the pollutant treatment enterprise decreases as the negotiation rounds increase. Therefore, SMEs prefer to bargain as late as possible in the negotiation sequence. However, the right to determine the negotiation sequence is owned by the pollutant treatment enterprise, who will force SMEs to pay for the position in the negotiation sequence. Then, SMEs should form a grand alliance to take part in the negotiation.

If $C_{e}^{\prime \prime }(\sum E_{i})<0$, the marginal pollutant treatment cost of the pollutant treatment enterprise is irrelative to the negotiation round. Therefore, SMEs will not pay for the position in the negotiation sequence, and it is unnecessary for them to form alliances.

**Proposition 9.**  If $C_{e}^{\prime \prime }(\sum E_{i})<0$, SMEs will take part in negotiation individually, and they will allocate profits with the pollutant treatment enterprise according to Proposition 4, and the pollutant treatment enterprise charges SMEs the tiered prices according to Proposition 5. If $C_{e}^{\prime \prime }(\sum E_{i})<0$, SMEs should form a grand alliance to take part in the negotiation, and they will allocate profits with the pollutant treatment enterprise according to Proposition 7, and the pollutant treatment enterprise charges SMEs the tiered prices according to Proposition 8.

**Proof of Proposition 9.**  The proof of Proposition 8 can be obtained through Propositions 4, 5, 7 and 8, and the above analysis.

## 4. Numerical Analyses

There are 100 SMEs of the same capability producing the same product at a selling price 50 per unit. The production cost function of all SMEs is *C_i_* = 0.05$q_{i}^{2}$, emission function is *E_i_* = 0.1*qi*, *i* = 1, 2, …, 100. There are two kinds of pollutant treatment cost functions of the pollutant treatment enterprise, which are respectively $C_{e}=10\sum E_{i}$ and $C_{e}=80\sqrt{\sum E_{i}}$. The revocation cost parameters of the pollutant treatment enterprise *e* and SME *i* are *k_e_* = 11 and *k_i_* = 3. Then, their bargaining powers are, respectively, *α_e,m_* = 0.75 and *β_e,m_* = 0.25, where *m* = *s*_1_, *i*.

If $C_{e}=10\sum E_{i}$, $C_{e}^{\prime }=10$, it means the marginal pollutant treatment cost is fixed. From Propositions 4, 5, 7, and 8, we can obtain that SMEs should take part in the negotiation individually. SMEs’ individual product quantity is $q_{i}^{*}=490$. The profits of the SME *i* and the pollutant treatment enterprise are, respectively, $\pi_{i}^{*}=3001.25$ and $\pi_{e}^{*}=9003.75,$ where $\pi_{e}^{*}$ is the profit that the pollutant treatment enterprise obtains from just one SME. The pollutant treatment enterprise charges SMEs a pollutant treatment fee per unit pollutant $P_{i}^{*}=193.75$.

If the pricing policy of the pollutant treatment enterprise is $P_{i}^{*}=193.75$, SME *i* produces ${\bar{q}}_{i}=306.25$, and the profits of the SME *i* and the pollutant treatment enterprise are ${\bar{\pi }}_{i}=4689.45$ and ${\bar{\pi }}_{e}=5627.35,$ respectively. SME *i* increases its profits at the price of the decrease of the total profits (decreasing from 12,005 to 10,316.8) and the pollutant treatment enterprise’s profit. Therefore, the pollutant treatment enterprise should charge SME *i* is $P_{i}=193.75E_{i}\left({\bar{q}}_{i}\right)+3376.41\text{ when }E_{i}\left({\bar{q}}_{i}\right)<E_{i}\left(q_{i}^{*}\right);\;P_{i}=193.75E_{i}\left({\bar{q}}_{i}\right)\text{ if }E_{i}\left({\bar{q}}_{i}\right)=E_{i}\left(q_{i}^{*}\right).$

With this pricing policy, the profits of the SME *i* and the pollutant treatment enterprise are respectively ${\bar{\pi }}_{i}=1313.05$ and ${\bar{\pi }}_{e}=9003.75$ if SME *i* still produces ${\bar{q}}_{i}=306.25,$ while they respectively obtain $\pi_{i}^{*}=3001.25$ and $\pi_{e}^{*}=9003.75$ if SME *i* produces $q_{i}^{*}=490.$ Therefore, SME *i* will produce $q_{i}^{*}=490$.

If $C_{e}=80\sqrt{\sum E_{i}}$, from Propositions 4 and 5, we can get that if SMEs take part in the negotiation individually, the product quantity and profits of the SME 1 (we take the first negotiation as example) are respectively $q_{i}^{*}=494.31$ and $\pi_{1}^{*}=2938.98$. The profits and the pricing policy of the pollutant treatment enterprise are respectively $\pi_{e}^{*}=8951.94$ and $P_{i}=192.48E_{i}\left({\bar{q}}_{i}\right)+3476.46\text{ when }E_{i}\left({\bar{q}}_{i}\right)<E_{i}\left(q_{i}^{*}\right);\;P_{i}=192.48E_{i}\left({\bar{q}}_{i}\right)\text{ if }E_{i}\left({\bar{q}}_{i}\right)=E_{i}\left(q_{i}^{*}\right).$

From Propositions 7 and 8, we can get that SMEs take part in the negotiation in a grand alliance, the product quantity and profits of SME *i* are, respectively, $q^{**}=499.43$ and $\pi_{i}^{**}=3100.86$. The profits and the pollutant treatment fee per unit pollutant of the pollutant treatment enterprise are, respectively, $\pi_{e}^{**}=9332.59$ and $P^{**}=188$.

If the pricing policy of the pollutant treatment enterprise is $P^{**}=188$, SME *i* produces $\tilde{q}=312,$ and the profits of SME *i* and the pollutant treatment enterprise are, respectively, $\pi_{i}^{*}=4867.35$ and $\pi_{e}^{*}=1396.95.$ Therefore, the pollutant treatment enterprise should charge SME *i* $P_{i}=188E_{i}\left(q_{i}\right)+7935.64\text{ when }E_{i}\left(q_{i}\right)<E_{i}\left(q_{i}^{**}\right);\;P_{i}=188E_{i}\left(q_{i}\right)\text{ if }E_{i}\left(q_{i}\right)=E_{i}\left(q_{i}^{**}\right).$ At this pricing policy, the profits of the SME *i* and the pollutant treatment enterprise are, respectively, ${\tilde{\pi }}_{i}=-3068.29$ and ${\tilde{\pi }}_{e}=-9332.59$ if SME *i* still produces $\tilde{q}=312$, while they obtain $\pi_{i}^{**}=3110.86$ and $\pi_{e}^{**}=9332.59,$ respectively, if SME *i* produces $q^{**}=499.43.$ Therefore, SME *i* will produce $q^{**}=499.43.$

From the above analyses, we can find that just as Proposition 9 points out that if $C_{e}^{\prime \prime }\left(\sum E_{i}\right)=0,$ SMEs should take part in the negotiation individually. Just as Propositions 5 and 6 point out that the pollutant treatment enterprise should design a mechanism, charging SMEs tiered prices, to stimulate SMEs to produce and emit the optimal quantities. Now, we analyze the change of the optimal cooperation policies when the selling price of product fluctuates.

Table 1 shows that with the increase of product selling price, it keeps rising that the optimal product quantity $q_{i}^{*},$ the optimal pollutant treatment fee per unit pollutant $P_{i}^{*}$and the maximal profits of SMEs and the pollutant treatment enterprise, $\pi_{i}^{*}$ and $\pi_{e}^{*}$. Meanwhile, SMEs earn more profits if they produce $q_{i}^{*}$ instead of ${\bar{q}}_{i}$, which means they will keep to their agreement with the pollutant treatment enterprise. Furthermore, the profits earned by SMEs through bargaining individually are the same as those through bargaining in alliance. Therefore, it is unnecessary for SMEs to form an alliance.

In Table 2, if SMEs bargain individually, and we still take the first negotiation as example, we can find that the profits earned by SMEs through bargaining individually are less than those through bargaining in alliance. Therefore, SMEs will form an alliance to bargain, and the pollutant treatment enterprise just needs to design a pricing mechanism for the SME alliance. Meanwhile, SMEs will keep to their agreement with the pollutant treatment enterprise, since they earn more profits if they produce $q^{**}$ instead of $\tilde{q}$ under this mechanism. From the above analyses, we can conclude that our model holds up against price fluctuations.

## 5. Conclusions

In this paper, we assume that a professional pollutant treatment enterprise provides a centralized pollutant treatment service for several SMEs, who can form alliances to negotiate with it. Then, we proposed a bargaining game model in this centralized pollutant treatment system to study the pollutant treatment enterprise’s pricing policies and SMEs’ alliance policies. Furthermore, we studied how the pollutant treatment enterprise prevents the moral hazard of SMEs producing products and emitting pollutants in breach of contract. The optimal solution on the price policy and SMEs’ alliance policy is obtained by theoretical and numerical analysis. It is found that the pollutant treatment enterprise can prevent SMEs’ moral hazard through tiered pricing. If the marginal treatment cost of the pollutant treatment enterprise is a constant, SMEs can maximize their profits by bargaining with the pollutant treatment individually. If the marginal treatment cost of the pollutant treatment enterprise decreases with the emission quantity, SMEs have to pay the pollutant treatment enterprise for the position in the negotiation sequence. Therefore, SMEs should form a grand alliance to negotiate as a whole to maximize their profits.

This paper focuses on the policy design for cooperation between SMEs and the professional pollutant treatment enterprise. However, the research methods and conclusions of this paper not only suit this case, but all the cases of centralized pollutant treatment as well. For example, centralized hazardous waste treatment models are implemented in many countries all over the world. In this case, a centralized hazardous waste treatment plant treats various kinds of hazardous wastes for the multiple manufacturers generating those wastes [31,32,33]. They need to determine the treatment price through negotiation. The main difference between this case and our case is that these manufacturers may be scattered far from each other, which results in the difficulty of government monitoring and the possibility of manufacturers’ stealthy emissions. Therefore, in consideration of government monitoring and regulation, our methods suit the case of centralized hazardous waste treatment. This will be the topic of our future research.

In this paper, we assume that the marginal pollutant treatment cost decreases as the quantity of pollutant emission increases. In some cases, however, the marginal pollutant treatment cost may increase with the increase of emission quantity. Therefore, the alliance policy of SMEs and pricing policy of the pollutant treatment enterprise in such a case are worth future study.

## Figures and Tables

**Table 1:** The optimal cooperation policies of different selling prices when $C_{e}=10\sum E_{i}$.

Selling Price	Ally or Not	$q_{i}^{*}$	$P_{i}^{*}$	$\pi_{i}^{*}$	$\pi_{e}^{*}$	${\bar{q}}_{i}$	${\bar{\pi }}_{i}$ when $q_{i}={\bar{q}}_{i}$	${\bar{\pi }}_{i}$ when $q_{i}=q_{i}^{*}$
40	No	390	156.25	1901.25	5703.75	243.75	831.80	1901.25
	Yes	390	156.25	1901.25	5703.75	243.75	831.80	1901.25
50	No	490	193.75	3001.25	9003.75	306.25	1313.05	3001.25
	Yes	490	193.75	3001.25	9003.75	306.25	1313.05	3001.25
60	No	590	231.25	4351.25	13053.80	368.75	1903.67	4351.25
	Yes	590	231.25	4351.25	13053.80	368.75	1903.67	4351.25
70	No	690	268.75	5951.25	17853.80	431.25	2603.67	5951.25
	Yes	690	268.75	5951.25	17853.80	431.25	2603.67	5951.25

**Table 2:** The optimal cooperation policies of different selling prices when $C_{e}=80\sqrt{\sum E_{i}}$.

Selling Price	Ally or Not	$q_{1}^{*}$ or $q^{**}$	$P_{1}^{*}$ or $P^{**}$	$\pi_{1}^{*}\;or\;\pi_{i}^{**}$	$\pi_{e}^{*}\;or\;\pi_{e}^{**}$	$\tilde{q}$	${\tilde{\pi }}_{i}\;when$ $q_{i}=\tilde{q}$	${\tilde{\pi }}_{i}\;when$ $q_{i}=q_{i}^{**}$
40	No	393.62	155.58	1874.01	5622.04	-	-	-
	Yes	399.37	150.55	1987.36	5962.07	249.45	−3090.96	1987.36
50	No	494.31	192.48	2983.98	8951.94	-	-	-
	Yes	499.43	188.00	3110.86	9332.59	312.00	−3068.29	3110.86
60	No	594.81	229.54	4345.42	13036.20	-	-	-
	Yes	599.48	225.45	4484.51	13453.50	374.55	−2890.99	4484.51
70	No	695.20	266.70	5957.95	17873.90	-	-	-
	Yes	699.08	262.92	6108.27	18324.80	437.08	−2570.09	6108.27

## Appendix

**Proof of Proposition 2.** Without loss of generality, we assume that SME *i*, who bargains at the (*j* − *ε*)*^th^* negotiation (*i* = 1, 2, …, *n*; *j* = 2, 3, …, *n*; *ε* ∈ {1, 2, …, *j* − 1}), takes part in the *j^th^* negotiation. From Equation (2), even if the product quantity and pollutant emission quantity of SME *i* at the *j^th^* negotiation equal those at the (*j* − *ε*)*^th^* negotiation, the difference of the total profit between the *j^th^* negotiation and the (*j* − *ε*)*^th^* negotiation is $\Pi_{j}-\Pi_{j-\varepsilon }=\left[C_{e}\left(Q_{j-\varepsilon }+E_{i}\right)-C_{e}\left(Q_{j-\varepsilon }\right)\right]-[C_{e}\left(Q_{j-\varepsilon }+\sum_{l=j-\varepsilon +1}^{j-1}E_{l}+E_{i}\right)-C_{e}(Q_{j-\varepsilon }+\sum_{l=j-\varepsilon +1}^{j-1}E_{l})]\geq 0$ because $C_{e}^{\prime \prime }(\sum^{\text{​}}E_{i})>0$ and $C_{e}^{\prime \prime }(\sum^{\text{​}}E_{i})\leq 0$. In other words, the total profits at the *j^th^* negotiation are, at least $\Pi_{j}-\Pi_{j-\varepsilon }$ higher than that at the (*j* − *ε*)*^th^* negotiation.

**Proof of Proposition 3.** As the total profit at the *j^th^* (*j* = 1, 2, …, *n*) negotiation is $\Pi_{j}^{*}-\Pi_{1}^{*}$ higher than that at the first negotiation, SME *i* (*i* = *j*) can get an extra profit of $\beta_{e,m}\left(\Pi_{j}^{*}-\Pi_{1}^{*}\right),$ where *m* = *s_j_*, by bargaining at the *j^th^* negotiation rather than at the first negotiation. Therefore, SME *i* prefers to bargain at the *j^th^* negotiation. However, as SME *i* has no right to determine the negotiation sequence, which is owned by the pollutant treatment enterprise, SME *i* has to make a payment to the pollutant treatment enterprise for the right of bargaining at the *j^th^* negotiation. As a matter of course, the upper limit of the payment is the profit difference between the *j^th^* negotiation and the first negotiation, $\Delta \pi_{i}^{*}=\beta_{e,m}\left(\Pi_{j}^{*}-\Pi_{1}^{*}\right)$, which is certainly the request of the pollutant treatment enterprise. As a result, SME *i* makes a payment $\Delta \pi_{i}^{*}=\beta_{e,m}\left(\Pi_{j}^{*}-\Pi_{1}^{*}\right)$ to the pollutant treatment enterprise.

**Proof of Proposition 4.** From Propositions 1 and 2, we can find that as SME *i* (*i* = *j* = 1, 2, …, *n*) makes a payment $\Delta \pi_{i}^{*}=\beta_{e,m}\left(\Pi_{j}^{*}-\Pi_{1}^{*}\right)$ to the pollutant treatment enterprise for the right of bargaining at the *j*^th^ negotiation, their final profits are $\beta_{e,m}\Pi_{j}^{*}-\Delta \Pi_{i}^{*}=\;\beta_{e,m}\Pi_{1}^{*},$ the pollutant treatment enterprise’s final profits are $\sum^{\text{​}}\left(\alpha_{e,m}\Pi_{j}^{*}-\Delta \Pi_{i}^{*}\right)=\sum \left(\Pi_{j}^{*}-\beta_{e,m}\Pi_{1}^{*}\right).$

**Proof of Proposition 5.** When the real emission quantity of SME *i** (i = j =* 1, 2, …, n*)* is less than the optimal one, namely, $E_{i}\left({\bar{q}}_{i}\right)<E_{i}\left(q_{i}^{*}\right),$ the pollutant treatment enterprise still charges SME *i* the treatment price per unit of pollutant $P_{i}^{*},$ and it obtains a profit $P_{i}^{*}E_{i}\left({\bar{q}}_{i}\right)-\left[C_{e}\left(Q_{j}+E_{i}\left({\bar{q}}_{i}\right)\right)-C_{e}\left(Q_{j}\right)\right],$ which is $F_{i}^{*}$ less than its optimal profit $P_{i}^{*}E_{i}\left(q_{i}^{*}\right)-\left[C_{e}\left(Q_{j}+E_{i}\left(q_{i}^{*}\right)\right)-C_{e}\left(Q_{j}\right)\right].$ Therefore, charging SME *i* a fixed fee $F_{i}^{*}$ makes it get its optimal profit. Meanwhile, as ${\bar{q}}_{i}\neq q_{i}^{*},$ the total profits of SME *i* and the pollutant treatment enterprise are less than the optimal total profits, which causes the profits of SME *i* to be less than the profits of it producing $q_{i}^{*}$. As a result, SME *i* will produce $q_{i}^{*}$ and emit $E_{i}\left(q_{i}^{*}\right),$ and both parties will obtain their optimal profits. When $E_{i}\left({\bar{q}}_{i}\right)=E_{i}\left(q_{i}^{*}\right),$ the pollutant treatment enterprise charging SME *i* the treatment price per unit pollutant $P_{i}^{*},$ SME *i* will produce $q_{i}^{*}$ and emit $E_{i}\left(q_{i}^{*}\right),$ and both parties will achieve their optimal profits. When $E_{i}\left({\bar{q}}_{i}\right)>E_{i}\left(q_{i}^{*}\right),$ the extra product quantity ${\bar{q}}_{i}-q_{i}^{*}$ makes SME *i* an extra profit $\Delta \pi_{i}=P\left({\bar{q}}_{i}-q_{i}^{*}\right)-\left[C_{i}\left({\bar{q}}_{i}\right)-C_{i}\left(q_{i}^{*}\right)\right]-{\bar{P}}_{i}\left[E_{i}\left({\bar{q}}_{i}\right)-E_{i}\left(q_{i}^{*}\right)\right].$ If the pollutant treatment enterprise charges SME *i* a treatment price per unit pollutant ${\bar{P}}_{i}>\frac{P\left({\bar{q}}_{i}-q_{i}^{*}\right)-\left[C_{i}\left({\bar{q}}_{i}\right)-C_{i}\left(q_{i}^{*}\right)\right]}{E_{i}\left({\bar{q}}_{i}\right)-E_{i}\left(q_{i}^{*}\right)}$ for the extra emissions $E_{i}\left({\bar{q}}_{i}\right)=E_{i}\left(q_{i}^{*}\right),$ the extra profits of SME *i* are negative, that is, $\Delta \pi_{i}<0.$ As a result, SME i will produce $q_{i}^{*}$ and emit $E_{i}\left(q_{i}^{*}\right),$ and both parties will get their optimal profits. Therefore, if the pollutant treatment enterprise charges SMEs the above tiered prices, they will produce and emit the optimal quantities, and both parties will obtain their optimal profits.

**Proof of Proposition 6.** From the analyses of Proposition 6 and Propositions 1 and 2 above, we can find that the pollutant treatment enterprise and SMEs alliance *s_j_* (*j* = 1, 2, …, *θ*), respectively obtain profits $\alpha \Pi_{j}^{**}$ and $\beta \Pi_{j}^{**}$ at the *j^th^* negotiation. SMEs *i* of alliance *s_j_* (*i* ∈ *s_j_*) obtain $\beta \Pi_{j}^{**}/\eta_{j}$ by allocating $\beta \Pi_{j}^{**}$ equally. Obviously, their profits are $\beta \left(\Pi_{j}^{**}-\Pi_{1}^{**}\right)/\eta_{j}$ more than the profits obtained bargaining at the first negotiation. However, the right of determining the negotiation sequence is owned by the pollutant treatment enterprise, so the SMEs alliance *s_j_* has to pay the pollutant treatment enterprise $\beta \left(\Pi_{j}^{**}-\Pi_{1}^{**}\right)$ for the right of bargaining at the *j^th^* negotiation, which costs the SMEs *i* of alliance $\beta \left(\Pi_{j}^{**}-\Pi_{1}^{**}\right)/\eta_{j}$. As a result, the pollutant treatment enterprise and SMEs *i* of alliance *s_j_* respectively obtain final profits $\Pi_{j}^{**}-\beta \Pi_{1}^{**}$ and $\beta \Pi_{1}^{**}/\eta_{j}.$

**Proof of Proposition 7.** When all SMEs form a grand alliance to participate in the negotiation, the pollutant treatment enterprise and all SMEs firstly determine the SMEs’ optimal product quantities. In this case, all SMEs produce the same quantity and obtain the same profit. Let $\Pi =\pi_{e}+n\pi_{i}$ and $q_{i}=q$, (*i* = 1, 2, …, *n*). Solving ∂∏/∂*q* = 0, we can get the optimal product quantity of SME *i q***, and the maximum total profit $\Pi^{**}=pnq^{**}-C_{e}\left(nE^{**}\right)-nC_{i}\left(q^{**}\right).$ Subsequently, they bargain to allocate $\Pi^{**},$ from which the pollutant treatment enterprise obtains $\alpha \Pi^{**}$ and SMEs grand alliance obtains $\beta \Pi^{**}$. Finally, SME *i* obtains $\beta \Pi^{**}/n$ by allocating $\beta \Pi^{**}$ equally. As $\Pi^{**}$ is the maximum profit in centralized pollutant treatment, and SME *i* (*i* = 1, 2, …, *n*) has to pay the pollutant treatment enterprise except when bargaining in a grand alliance, $\beta \Pi^{**}/n$ is the maximum profit of SME *i*. Therefore, all SMEs should form a grand alliance to take part in the negotiation, in which the pollutant treatment enterprise and SMEs *i* obtain final profits $\alpha \Pi^{**}$ and $\beta \Pi^{**}/n,$ respectively.

**Proof of Proposition 8.** When the real emission quantity of SME *i* (*i* = *j* = 1, 2, …, *n*) is less than the optimal one, namely, $E_{i}\left(\tilde{q}\right)<E_{i}\left(q^{**}\right),$ the pollutant treatment enterprise still charges SME *i* the treatment price per unit pollutant *P***, it obtains profits $P^{**}E_{i}\left(\tilde{q}\right)-\frac{C_{e}\left(nE_{i}\left(\tilde{q}\right)\right)}{n}$ which is *F*** less than its optimal profits $P^{**}E_{i}\left(q^{**}\right)-\frac{C_{e}\left(nE_{i}(q^{**}\right)}{n}$. Therefore, charging SME *i* a fixed fee *F*** makes it obtain its optimal profits. Meanwhile, as $\tilde{q}<q^{**},$ the total profits of SME *i* and the pollutant treatment enterprise are less than the optimal total profits, which causes the profits of SME *i* to be less than its profits of it were producing *q***. As a result, SME *i* will produce *q*** and emit $E_{i}\left(q^{**}\right),$ and both parties will get their optimal profits. When $E_{i}\left(\tilde{q}\right)=E_{i}\left(q^{**}\right)$, the pollutant treatment enterprise charges SME *i* the treatment price per unit pollutant *P***, SME *i* will produce *q*** and emit $E_{i}\left(q^{**}\right)$, and both parties will attain their optimal profits. When $E_{i}\left(\tilde{q}\right)>E_{i}\left(q^{**}\right)$, the extra product quantity $\tilde{q}-q^{**}$ makes SME *i* an extra profit $\Delta \pi_{i}=P\left(\tilde{q}-q^{**}\right)-\left[C_{i}\left(\tilde{q}\right)-C_{i}\left(q^{**}\right)\right]-\tilde{P}\left[E_{i}\left(\tilde{q}\right)-E_{i}\left(q^{**}\right)\right].$ If the pollutant treatment enterprise charges SME *i* a treatment price per unit pollutant $\tilde{P}>\frac{P\left(\tilde{q}-q^{**}\right)-\left[C_{i}\left(\tilde{q}\right)-C_{i}\left(q^{**}\right)\right]}{E_{i}\left(\tilde{q}\right)-E_{i}\left(q^{**}\right)}$ for the extra emissions $E_{i}\left(\tilde{q}\right)-E_{i}\left(q^{**}\right)$, the extra profits of SME *i* are negative, that is, $\Delta \pi_{i}<0$. As a result, SME *i* will produce $q^{**}$ and emit $E_{i}\left(q^{**}\right)$, and both parties will get their optimal profits. Therefore, if the pollutant treatment enterprise charges SMEs the above tiered prices, they will produce and emit the optimal quantities, and both parties will get their optimal profits.

## References

[1] Agan Y., Acar M.F., Borodin A. (2013). Drivers of environmental processes and their impact on performance: A study of Turkish SMEs. J. Clean. Prod..

[2] Li Y.Y., Huang B. (2013). Multiple Principal-Agent Model of SMEs Pollution Regulation under Centralized Treatment. J. Netw..

[3] Zeng X., Meng H., Zeng R.C., Tam C.M., Tam V.W.Y., Jin T. (2011). How environmental management driving forces affect environmental and economic performance of SMEs: A study in the Northern China district. J. Clean. Prod..

[4] Stevens R.J.L., Moustapha M.M., Evelyn P., Stevenson R.J. (2013). Analysis of the Emerging China Green Era and Its Influence on Small and Medium-Sized Enterprises Development: Review and Perspectives. J. Sustain. Dev..

[5] Studer S., Welford R., Hills P. (2006). Engaging Hong Kong Businesses in Environmental Change: Drivers and Barriers. Bus. Strateg. Environ..

[6] Tanaka M. (2012). Multi-Sector Model of Tradable Emission Permits. Environ. Resour. Econ..

[7] Chen H.C., Liu S.M., Chang C.Y. (2013). Commitment or no-commitment to monitoring in emission tax systems?. Environ. Econ. Policy Stud..

[8] Paetzold A., Smith M., Warren P.H., Maltby L. (2011). Environmental impact propagated by cross-system subsidy: Chronic stream pollution controls riparian spider populations. Ecology.

[9] Masurel E. (2007). Why SMEs invest in environmental measures: Sustainability evidence from small and medium-sized printing firms. Bus. Strateg. Environ..

[10] Jansson J., Nilsson J., Modig F., Vall G.H. (2015). Commitment to Sustainability in Small and Medium-Sized Enterprises: The Influence of Strategic Orientations and Management Values. Bus. Strateg. Environ..

[11] Brammer S., Hoejmose S., Marchant K. (2012). Environmental Management in SMEs in the UK: Practices, Pressures and Perceived Benefits. Bus. Strateg. Environ..

[12] Hargreaves J.J., Hobbs B.F. (2009). Optimal Selection of Priority Development Areas Considering Tradeoffs between Hydrology and Development Configuration. Environ. Model. Assess..

[13] Roomratanapun W. (2001). Introducing centralised wastewater treatment in Bangkok: A study of factors determining its acceptability. Habitat Int..

[14] Yuan Z.W., Jiang W.L., Bi J. (2010). Cost-effectiveness of two operational models at industrial wastewater treatment plants in China: A case study in Shengze town, Suzhou City. J. Environ. Manag..

[15] Chittock D.G., Hughey K.F.D. (2011). A review of international practice in the design of voluntary pollution prevention programs. J. Clean. Prod..

[16] Avram D.O., Kühne S. (2008). Implementing responsible business behavior from a strategic management perspective: Developing a framework for Austrian SMEs. J. Bus. Ethics.

[17] He Y., He A.Y. (2007). Analysis on Impact of SMEs’ Character on Pollution Control and Its Solution. China Econ..

[18] Hu X.P., Huang B. (2012). Regulation Policy of SMEs’ Pollutant Emission under Centralized Pollution Control. Mod. Manag. Sci..

[19] Li Y.Y. (2012). Research on Environment Regulation Policy for SMEs. Mod. Manag. Sci..

[20] Cui Z.F., Meng W.D., Liu J.P. (2011). Pricing Model of Pollutant Emission for Small-and-Medium-Size Enterprises under Centralized Pollution Control Mode. Ind. Eng. J..

[21] Nash J. (1953). Two-person cooperative games. Econom. J. Econom. Soc..

[22] McGuire T.W., Staelin R. (1983). An industry equilibrium analysis of downstream vertical integration. Mark. Sci..

[23] Nagarajan M., Bassok Y. (2008). A bargaining framework in supply chains: The assembly problem. Manag. Sci..

[24] Escapa M., Gutiérrez M.J. (1997). Distribution of Potential Gains from International Environmental Agreements: The Case of the Greenhouse Effect. J. Environ. Econ. Manag..

[25] Solow J.L., Krautmann A.C. (2011). A Nash Bargaining Model of the Salaries of Elite Free Agents. J. Sports Econ..

[26] Du J.Y. (2005). Effects of Price War and Strategies of Enterprises against the Price War. Econ. Probl..

[27] Park J.M., Park J.Y., Park H.S. (2016). A review of the National Eco-Industrial Park Development Program in Korea: Progress and achievements in the first phase, 2005–2010. J. Clean. Prod..

[28] O’Connor J.R., Citarella J.F. (1970). An air pollution control cost study of the steam-electric power generating industry. J. Air Pollut. Control Assoc..

[29] Wu D., Xu Y., Zhang S. (2015). Will joint regional air pollution control be more cost-effective? An empirical study of China’s Beijing–Tianjin–Hebei region. J. Environ. Manag..

[30] Muthoo A. (1996). A bargaining model based on the commitment tactic. J. Econ. Theory.

[31] Amsoneit N. (1996). A centralized hazardous waste treatment plant: The facilities of the ZVSMM at Schwabach as an example. Water Sci. Technol..

[32] Sun N., Wu S.Z., Hou G.G., Wang Q., Chen L., Sun Y.R. (2009). Construction Status, Problem and Measures of Integrated Facilities for Centralized Treatment and Disposal of Hazardous Wastes. Environ. Sci. Manag..

[33] Duan H., Huang Q., Wang Q., Zhou B., Li J. (2008). Hazardous waste generation and management in China: A review. J. Hazard. Mater..

